# Soil Carbon Stocks Decrease following Conversion of Secondary Forests to Rubber (*Hevea brasiliensis*) Plantations

**DOI:** 10.1371/journal.pone.0069357

**Published:** 2013-07-19

**Authors:** Marleen de Blécourt, Rainer Brumme, Jianchu Xu, Marife D. Corre, Edzo Veldkamp

**Affiliations:** 1 Soil Science of Tropical and Subtropical Ecosystems, Büsgen Institute, Georg-August-Universität Göttingen, Göttingen, Germany; 2 World Agroforestry Centre, East Asia Node, Kunming, China; DOE Pacific Northwest National Laboratory, United States of America

## Abstract

Forest-to-rubber plantation conversion is an important land-use change in the tropical region, for which the impacts on soil carbon stocks have hardly been studied. In montane mainland southeast Asia, monoculture rubber plantations cover 1.5 million ha and the conversion from secondary forests to rubber plantations is predicted to cause a fourfold expansion by 2050. Our study, conducted in southern Yunnan province, China, aimed to quantify the changes in soil carbon stocks following the conversion from secondary forests to rubber plantations. We sampled 11 rubber plantations ranging in age from 5 to 46 years and seven secondary forest plots using a space-for-time substitution approach. We found that forest-to-rubber plantation conversion resulted in losses of soil carbon stocks by an average of 37.4±4.7 (SE) Mg C ha^−1^ in the entire 1.2-m depth over a time period of 46 years, which was equal to 19.3±2.7% of the initial soil carbon stocks in the secondary forests. This decline in soil carbon stocks was much larger than differences between published aboveground carbon stocks of rubber plantations and secondary forests, which range from a loss of 18 Mg C ha^−1^ to an increase of 8 Mg C ha^−1^. In the topsoil, carbon stocks declined exponentially with years since deforestation and reached a steady state at around 20 years. Although the IPCC tier 1 method assumes that soil carbon changes from forest-to-rubber plantation conversions are zero, our findings show that they need to be included to avoid errors in estimating overall ecosystem carbon fluxes.

## Introduction

Deforestation and forest degradation in the tropics have been estimated to contribute 12–15% of the global anthropogenic CO_2_ emissions [1]. The majority of land-use induced CO_2_ emissions arise from the loss of above-ground biomass and to a lesser extent from decomposition of soil organic carbon [2], [3]. Currently, the estimations of land-use change effects on above-ground carbon stocks are improving due to remote sensing techniques. However, estimates of land-use change effects on soil carbon stocks remain inconclusive [4], [5].

In tropical regions, the magnitude and direction of land-use induced changes in soil carbon stocks are largely determined by mean annual rainfall and clay mineralogy [5], [6]. A large number of studies exist on the impact of tropical land-use changes on soil carbon stocks, especially on the conversion from forest to pasture, pasture to secondary forest, and forest to cropland [5]. However, the published field observations are unequally distributed over the tropics with regards to an area-weighted distribution of the above-mentioned biophysical conditions. In addition, little research has been done on currently important land-use changes, one of which is forest-to-rubber (*Hevea brasiliensis*) plantation conversion. These limitations in available field-observations hamper the estimates of land-use change effects on soil carbon stocks in the tropics [5].

Our present study focuses on the land-use change from secondary forests to rubber plantations in Xishuangbanna, the southernmost prefecture of Yunnan Province in the southwest of China. The area of monoculture rubber plantations is rapidly expanding in the montane mainland southeast Asia, spanning southwest China, Laos, Cambodia, Myanmar, northeast Thailand, and northwest Vietnam, causing a large decrease in the region’s forest cover [7]. Rubber trees were traditionally not grown in this region, since environmental conditions (low temperatures in winter and a distinctive dry season) were considered marginal for rubber trees [8]. The first rubber plantations were successfully established in Xishuangbanna by the Chinese government in the late 1950s, and the subsequent expansion of rubber plantations resulted in a strong economic development [9]. At present, rubber plantations in montane mainland southeast Asia cover an area of more than 1.5 million ha [8] of which 424,000 ha is in Xishuangbanna [10]. By 2050 the area of rubber plantations is predicted to increase fourfold, mainly by replacing secondary forests, and swidden-related bushes and shrublands [11].

Forest-to-rubber plantation conversion is an important recent land-use change for which environmental impacts have hardly been studied. Meta-analyses of current data have shown that changes of soil carbon stocks after conversion of forests to tree plantations are variable: no effects were reported for tropical tree plantations [5], [12] whereas in another review 0–30% decrease in soil carbon stocks were reported for intensified rubber plantations compared to swidden fields in southeast Asia [13]. However, this review was based on studies which did not have a clear reference land-use type for the rubber plantations but merely compared land-use types and therefore any detected difference cannot directly be attributed to changes in land use. To our knowledge there are only three published tropical studies that investigated the effects of forest-to-rubber plantation conversions on soil carbon stocks. Two out of the three studies focused on the conversion from primary forests to rubber plantations in Brazil [14], [15] and both reported declines in the soil carbon stocks in 17- and 22-year-old plantations. The other study focused on the conversion from secondary forests to rubber plantations and happened to be conducted in Xishuangbanna [16]. Yang et al. [16] reported a 20% decline in the soil carbon stock in the top 0.6-m depth in a 3-year-old plantation and a 16% decline in a 7-year-old plantation but these estimates were not corrected for changes in soil bulk density with land-use change. It is also important that studies include older rubber plantations as soil carbon stocks may reach equilibrium after several decades, and older plantations will allow us to quantify the long-term effects of this land-use change. Soil carbon losses after deforestation are often related to (i) changes in the quality and quantity of soil carbon input, (ii) accelerated soil carbon decomposition rates driven by changes in microclimatic conditions or the breakdown of soil aggregates, and (iii) enhanced soil surface erosion. The magnitude of soil carbon losses depends furthermore on site-specific conditions such as soil texture, soil mineralogy, topography and climate.

Improved estimates of the effects of this important land-use change on soil carbon stocks are essential to the national greenhouse gas inventories from the Conference of Parties of the United Nations Framework of Climate Change. Although the Intergovernmental Panel of Climate Change (IPCC) provides guidelines for the estimations of the ecosystem carbon fluxes arising from land-use changes, the IPCC tier 1 method assumes soil carbon changes to be zero for the conversion from forests to rubber plantations because of limited scientific knowledge [4], [17].

We conducted the present study in Xishuangbanna, Yunnan Province, China using a space-for-time substitution approach. Our objectives were: (i) to quantify changes in soil carbon stocks following conversion of secondary forests to rubber plantations over a 46 years’ time period, and (ii) to determine the biophysical factors which control soil carbon concentrations, and soil carbon stock changes. We hypothesized that conversion from secondary forests to rubber plantations would result in a decrease of soil carbon stocks. This decrease is expected to be related to management practices commonly employed in rubber plantations such as terrace construction and removal of the vegetation understory.

## Materials and Methods

### Study Area and Site Characteristics

**Figure 1:**
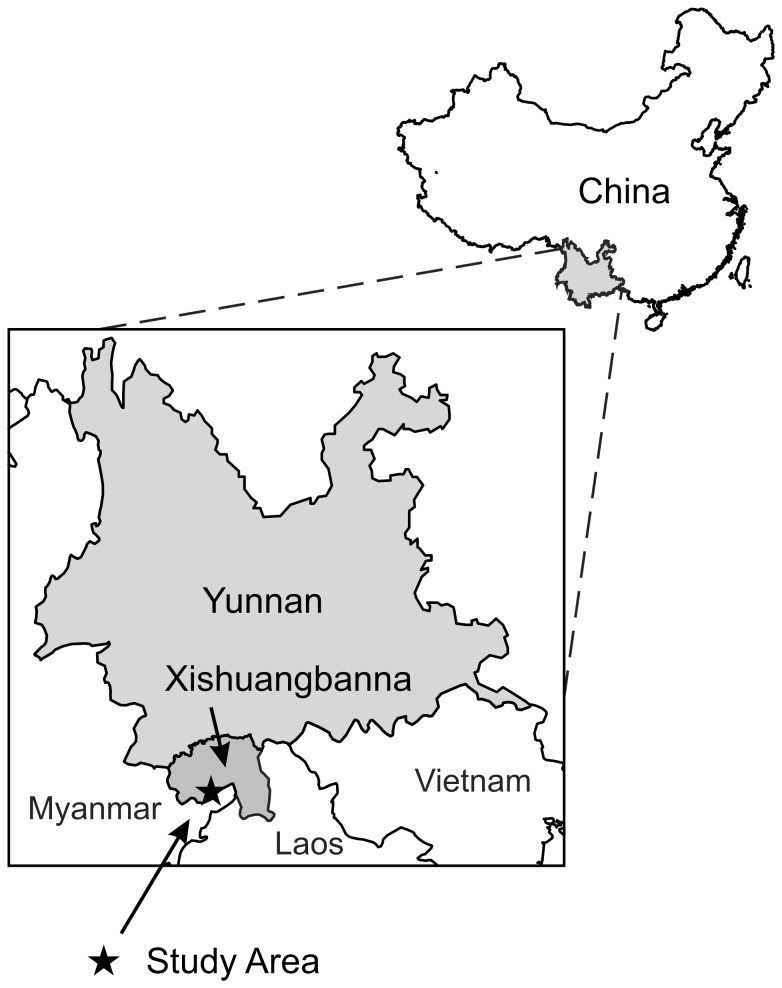
Location of the study area in Xishuangbanna prefecture, Yunnan province, China.

The study area of 4500 ha was located in Menglong township, Jinghong county of Xishuangbanna prefecture in Yunnan province, China (21°31′17.03″N, 100°37′12.13″E) (Fig. 1). The climate is tropical monsoon and is characterized by a dry season from November to April and a wet season from May to October. The mean annual rainfall is 1377 mm and the mean annual temperature is approximately 22.7°C [9]. The topography is hilly to mountainous, with an elevation that varies between 650 and 1450 m above sea level [9]. The study plots were located between 700 and 830 m above sea level. The soils at the plots are dominated with low activity clays and were classified as Ferralsols having an effective cation exchange capacity (ECEC) of less than 12 cmol_c_ kg^−1^ clay and as (hyper) ferralic Cambisols with an ECEC of less than 24 cmol_c_ kg^−1^ clay [18] (Table 1, Table S1A, Table S1B).

**Table 1:** Means^1^ (± SE) of soil characteristics of land-use types.

Characteristic	Depth (m)	Rubber plantation (n = 11)	Secondary forest (n = 7)
Sand (%)	0–0.15	32.5±3.8	34.7±4.6
	0.15–0.3	30.5±3.9	34.4±5.0
	0.3–1.2	27.8±3.4	30.2±4.4
Silt and clay (%)	0–0.15	67.5±3.8	65.3±4.6
	0.15–0.3	69.5±3.9	65.6±5.0
	0.3–1.2	72.2±3.4	69.8±4.4
Bulk density (g cm^−3^)	0–0.15	1.1±0.1	1.0±0.1
	0.15–0.3	1.1±0.0	1.2±0.0
	0.3–1.2	1.3±0.0	1.3±0.0
C:N ratio	0–0.15	12.2±0.3	12.8±0.5
	0.15–0.3	12.1±0.4	12.5±0.4
	0.3–1.2	9.5±0.4	9.7±0.5
pH (H_2_O)^2^	0–0.15	4.8±0.1 a	4.7±0.1 b
	0.15–0.3	4.8±0.1	4.9±0.1
	0.3–1.2	5.0±0.1	4.9±0.1
pH (KCl)	0–0.15	3.9±0.0	3.9±0.0
	0.15–0.3	3.8±0.1	3.9±0.0
	0.3–1.2	4.0±0.0	4.0±0.0
Effective cation exchangecapacity^2^ (mmol_c_ kg^−1^ soil)	0–0.15	46.4±1.8 a	55.5±2.4 b
	0.6–0.9	28.8±1.2 a	36.9±3.9 b
Base saturation (%)	0–0.15	24.4±4.2	24.1±6.0
	0.6–0.9	10.9±1.6	11.3±1.5

1 Means of the 0.3–1.2 m depth interval are means of the 0.3–0.6-m, 0.6–0.9-m and 0.9–1.2-m depth intervals.

2 Within a row, means followed by different letters differ significantly between rubber plantation and secondary forest (linear mixed effects model at P≤0.05).

### Current and Past Land Use

The major land-use types in Menglong township include rubber plantations, secondary forests, and farmland. According to local plantation owners the dominant land-use change trajectories in Menglong township were: (i) Primary forest–swidden agriculture–secondary forest–rubber plantation, and (ii) primary forest–swidden agriculture–rubber plantation. Swidden agriculture was the dominant land-use type in the region for centuries [19]; this involved cutting and burning of vegetation patches in the forest, thereby creating fields for use as rotation of cropping phases (1–3 years) and fallow periods (5–20 years) during which secondary vegetation regrows [11], [20]. The widespread practice of swidden cultivation in the past resulted in loss and degradation of primary forests [21]. Nowadays, almost all swidden fields have been replaced by monoculture rubber plantations. Since primary forest and swidden agriculture are not present anymore, we focused on the more recent land-use change from secondary forest towards rubber plantations. Based on information from local plantation owners, we selected rubber plantations that all went through this land-use change trajectory. Forest clearing was done by hand and no heavy machinery was used. After forest clearing, the sites were usually burnt. During the first four years after planting, rubber trees may have been intercropped with maize, upland rice, peanuts, and beans. In our study area, four forest remnants remain, including three collective forests and one “watershed protection” forest, which we used as our reference. These broadleaf forests are highly degraded due to the collection of firewood at present and timber extraction in the past. The forests have been cleared from primary forest, after which they were used for swidden agriculture and finally secondary forests were established (information from local farmers). The age of each forest remnant was estimated between 40–60 years. The size of the forest patches ranges from 20–60 ha.

### Management Practices in Rubber Plantations

Management practices commonly applied in rubber plantations are terrace establishment and maintenance, fertilizer application, pest control, management of the vegetation understory and rubber tapping. The terrace benches are constructed by hand using a hoe, cutting soil from upper parts of the slope and moving it to lower parts. During terrace maintenance, weeds are removed from the terrace risers by scraping off the soil, which is subsequently evenly distributed over the entire terrace bench. This process is repeated once or twice per year, resulting in older plantations having wider and deeper incised terraces. Rubber trees are planted in a row on the terrace benches and have a tree spacing ranging from 2–3 m. The step height of each individual terrace ranges from 0.3–0.8 m and bench width is typically ∼2.5 m. The horizontal distance between two adjacent terrace benches is 5–7 m, depending on the slope of the hill. Between some of the rubber trees pits are dug which have various uses: (i) as a measure to reduce runoff and retain soil moisture, and (ii) to apply fertilizer and collect leaf litter and cut herbs. The dimensions of these pits vary per plantation and range from: 0.4–1.3-m length × 0.2–0.5-m width × 0.2–0.35-m depth. Chemical fertilizers (NPK-compounds) are applied one to two times per year. The management of vegetation understory varies per rubber plantation; some plantation owners use herbicides to control the vegetation understory while others cut the vegetation understory back. Rubber tapping is usually done between April and October and latex collection is done every second day.

### Sampling Design

We used the space-for-time substitution approach to quantify changes in soil carbon stocks following conversion from secondary forests to rubber plantations. Soil carbon stocks were measured in clusters consisting of one reference secondary forest plot and one to three plots in rubber plantations. Clusters were established around randomly selected secondary forest plots. To avoid edge effects, the forest plots were selected at least 20 m from the forest edge. Within each cluster, the rubber plantations were chosen based on biophysical conditions, land-use history and distance to the reference plot. We only selected rubber plantations that were established immediately after forest clearing. To keep biophysical conditions within a cluster as similar as possible, we selected rubber plantations with similar altitude, slope, aspect, soil color and soil texture as the reference plot. The maximum distance between plots within a cluster was 3 km. In total we selected seven clusters, with a total of 11 rubber plantations and seven reference forests. The rubber plantations within each cluster differed in age ranging from 5 to 46 years. Selected rubber plantations were both state-owned rubber plantations and plantations belonging to smallholder farmers.

A critical assumption of the space-for-time substitution approach is that plots within a cluster were initially similar with regard to soil characteristics, soil carbon stocks, and land-use history such that measured differences in soil carbon stocks between the reference land-use type and the converted land-use type can be attributed to recent land-use change [22]. To test this assumption we compared land-use independent soil characteristics (i.e. soil texture) of plots within a cluster. Since we did not detect significant differences in soil texture between the secondary forest and rubber plantations within a cluster (Table 1), we assumed that the soils were originally similar and that observed soil carbon changes can be attributed to changes in land use.

### Fieldwork Permission

Our research was part of the project “Making the Mekong Connected (MMC)”. This project had been officially registered at the Kunming Institute of Botany, Chinese Academy of Sciences, which provided rights for access to field research in China. We received permission from the owners and managers of the rubber plantations to conduct the described fieldwork in their plantations. The secondary forests are part of local collective forests belonging to the villages. The local forestry station of Menglong Township, Jinghong County, has been informed a priori about our fieldwork in the secondary forests. No official permits were required for the described fieldwork since the secondary forests are not part of a national nature reserve. The fieldwork did not involve endangered or protected species.

### Plot Layout and Soil and Litter Sampling

In each land-use type, we established a plot with a size of 20×20 m, corrected for slope. Within each plot we established five parallel transects with 5-m spacing in between. Transects had a fixed north-south orientation. We randomly positioned four sampling points along each transect, resulting in 20 sampling points per plot.

Soil samples were collected down to 1.2-m depth from five depth intervals: 0–0.15 m, 0.15–0.3 m, 0.3–0.6 m, 0.6–0.9 m, and 0.9–1.2 m. The upper three depth intervals were sampled using a Dutch auger at the 20 sampling points. The 20 soil samples were pooled in the field to form one composite sample for each depth interval. Soil samples for the 0.6–0.9-m and 0.9–1.2-m depth were sampled in a soil pit which was positioned on the slope between two adjacent terraces in the middle of each plot. The soil samples were air dried for five days and sieved through a 2-mm sieve prior to laboratory analyses. Bulk-density samples were taken in the soil pit for each of the five depth intervals, using the core method [23]. Very few of the bulk-density samples contained stones or rock fragments and thus we did not correct for the gravel content.

Litter-layer samples were taken from every second sampling point, resulting in 10 litter samples per plot. Leaf litter and organic material (including seeds and twigs) were collected from a 0.04-m^2^ quadrant sample frame. The collected material was oven dried at 60°C for 48 hours and weighed. Subsamples of each sample were pooled by plot and analyzed for total carbon and nitrogen concentration. The carbon stock of the litter layer was calculated with the carbon concentration (%), the mass of the litter layer, and the sample-frame area.

### Tree Inventory, Topographical and Land use Data

In the rubber-plantation plots, we measured for all the trees the diameter at breast height (DBH) at 1.3 m above the soil surface. In the forest plots, we measured the DBH for trees with a DBH >4 cm, and the DBH of bamboos. For bamboos, we measured one stem DBH per clump and we recorded the number of stems per clump. Here we report both the tree basal area and total basal area, which is the sum of the basal area of trees and bamboos. Other site characteristics that were collected of each plot included: slope, aspect, altitude, and GPS coordinates. Information on current and past land use and their management was collected through interviews with land-owners and elders in the villages.

### Laboratory Analyses and Soil Carbon Stock Calculations

Total carbon and nitrogen concentrations were measured from ground soil and litter samples by dry combustion using CNS Elemental analyzer (Elementar Vario EL, Hanau, Germany). As soil pH was below 5.5, carbonates were not expected in these soils and we made no attempt to remove them. Soil pH (H_2_O) and pH (KCl) were measured on air-dried soil for all individual soil samples in a 1∶2.5 soil-to-solution ratio. ECEC was measured on soil samples of the 0–0.15-m and 0.6–0.9-m depth. The soil samples were percolated with un-buffered 1 M NH_4_Cl and the percolates were analyzed for exchangeable cations using ICP-AES (Spectroflame, Spectro Analytical Instruments, Kleve, Germany) [24]. Soil texture analyses were determined for all depth intervals with the pipette method, distinguishing the fractions: clay (<0.002 mm), silt (0.002–0.063 mm), and sand (0.063–2 mm).

Soil carbon stocks (Mg C ha^−1^) in each depth interval were calculated using the following equation:
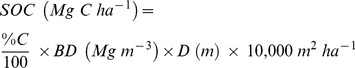
(1)where, BD is the bulk density and D refers to the thickness of the depth interval. Total soil carbon stocks down to 1.2-m depth were calculated as the sum over all depth intervals. Land-use changes often coincide with changes in bulk density due to management practices which may compact or loosen the soil. In order to be able to compare the same soil mass and to avoid the interference of bulk density changes with soil carbon stocks changes, we used the bulk density data of the reference plots to calculate the soil carbon stock of the rubber plantation plots [6].

### Statistical Analyses

All statistical analyses were done using the open source statistical software R version 2.15.0 [25]. To make statistical inferences on the differences in soil carbon stocks and soil characteristics between rubber plantations and secondary forest, we applied linear mixed effects models (LME) using the nlme package [26]. Response variables were the soil carbon stocks and soil characteristics and we included land-use type, depth interval, and the interaction between land-use type and depth interval as fixed effects. Cluster was included as a random factor. Comparisons of soil carbon stocks and soil characteristics between land-use types at each depth interval were obtained by defining and testing contrasts with the generalized linear hypothesis test in the multcomp package [27]. For the multiple comparisons of soil carbon stock changes between depth intervals, the P values were adjusted using Holm’s correction. For each LME, assumptions on normality and homogeneity of variance were checked by visual inspection of plots of residuals against fitted values. In cases of unequal variances of residuals, we included a variance function that allows for unequal variances [28].

To examine monotonic trends of soil carbon concentrations and relative soil carbon stock differences with potential explanatory variables, we did spearman rank correlation tests. Relative soil carbon stock differences were calculated as carbon stock in rubber plantation minus carbon stock of the reference secondary forest divided by carbon stock of the reference secondary forest multiplied by 100. Relative soil carbon stock differences were correlated with explanatory variables of the rubber plantations. As potential explanatory variables we included litter carbon stock, litter C:N ratio, total basal area, sum of silt and clay content, slope, and altitude. Correlation tests were done for each depth interval.

The trend between soil carbon and rubber plantation age was examined using non-linear regression. We tested the fit of both a mono-exponential model and a bi-exponential model according to Lobe et al. [29]. The mono-exponential model assumes a single soil carbon pool which following land-use change tends towards a new equilibrium:
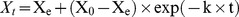
(2)where, X_0_ is the initial soil carbon stock of the secondary forest plots (t = 0), X_t_ is the soil carbon stock in the rubber plantation plots at age t, X_e_ is the soil carbon stock at steady state, k is the decay rate per year, and t is year since land-use change. The age t at which steady state was reached was calculated as the point where the proportion of carbon remaining in the soil (X_t_) did not differ more than 1% of the calculated steady state value X_e_ [29]. The bi-exponential model considers both labile and stable soil carbon pools:
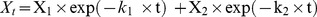
(3)where, X_1_ is the proportion of carbon in the labile pool, and X2 is the proportion of carbon in stable pool (X_2_ = 100– X_1_), k_1_ is the decay rate per year of the labile pool, k_2_ is the decay rate per year of the stable pool. We expressed soil carbon as the proportion of the soil carbon stock in the rubber plantation to the initial amount in the reference secondary forest. The exponential models were fitted to the data using nonlinear least-squares estimations. The goodness of the fit was assessed by Pearson’s correlation coefficient (r) showing the relationship between the observed and fitted values.

## Results

### Soil Characteristics, Litter Layer, and Tree Basal Area in Rubber Plantations and Secondary Forests

Soil texture, bulk density, soil C:N ratio, pH (KCl), and base saturation did not differ between rubber plantations and secondary forests (Table 1). The pH (H_2_O) in the top 0.15-m depth was higher in rubber plantations than in secondary forests. The ECEC in all depth intervals was lower in rubber plantations than in secondary forest. Litter carbon concentration, litter C:N ratio, and litter carbon stock did not differ between rubber plantations and secondary forests (Table 2). The tree basal area in rubber plantations ranged from 3.2 to 42.4 and was positively correlated with plantation age (spearman’s rho = 0.93, p≤0.001); the mean tree basal area was twice that of the secondary forests (Table 2). However, the total basal area (sum of trees and bamboos) did not differ between rubber plantations and secondary forests.

**Table 2:** Means (± SE) of litter and tree characteristics of land-use types.

Characteristic	Rubber plantation (n = 11)	Secondary forest (n = 7)
Litter carbon concentration (%)	41.1±0.7	40.0±0.7
Litter C : N ratio	46.1±3.8	44.9±3.6
Litter carbon stock (Mg C ha^−1^)	2.1±0.2	2.7±0.4
Tree basal area^1^ (m^2^ ha^−1^)	18.6±3.8 a	9.7±2.4 b
Total basal area^2^ (m^2^ ha^−1^)	18.6±3.8	15.3±1.7

1 Within a row, means followed by different letters differ significantly between rubber plantation and secondary forest (linear mixed effects model at P≤0.05).

2 Total basal area is calculated as the sum of the basal area of trees and bamboos.

### Soil Carbon Concentrations and Stocks in Rubber Plantations and Secondary Forests

All rubber plantations had a lower soil carbon stock in the total soil profile (0–1.2-m depth) than secondary forests (P≤0.01) (Table 3). The differences in soil carbon stocks between rubber plantations and secondary forests ranged from −15.4 to −59.4 Mg C ha^−1^ with a mean of −37.4±4.7 Mg C ha^−1^, equivalent to a 19.3±2.7% loss of the initial soil carbon stock. The soil carbon losses were depth dependent as was shown by a significant interaction between land-use type and soil depth (P≤0.001). The decrease in soil carbon concentrations and soil carbon stocks was significant for the three depth intervals in the top 0.6-m depth (Table 3). The largest decrease was found in the top 0.15-m of the soil (P≤0.01) accounting for 32% of soil carbon losses.

**Table 3:** Means (± SE) of soil carbon stocks and absolute^1^ and relative^2^ differences between land-use types.

	Rubber plantation (n = 11)		Secondary forest (n = 7)		Difference (n = 7)	
Depth (m)	C (%)	C (Mg ha^−1^)	C (%)	C (Mg ha^−1^)	Absolute (Mg C ha^−1^)	Relative (C %)
0–0.15	2.1±0.1	30.3±1.9	2.9±0.1	43.9±2.6	−11.8±1.1***	−26.9±2.8***
0.15–0.3	1.7±0.1	29.8±1.6	2.2±0.1	38.9±1.5	−8.2±1.4 ***	−21.4±3.2***
0.3–0.6	1.2±0.1	43.6±2.6	1.4±0.1	52±1.6	−8.0±3.0*	−15.4±5.6*
0.6–0.9	0.7±0.1	28.0±1.9	0.9±0.1	35.2±3.7	−6.5±3.6	−16.0±8.0
0.9–1.2	0.6±0.0	23.2±1.3	0.7±0.0	26.0±1.0	−2.9±1.8	−11.2±7.0
Total	–	154.9±6.2	–	196.0±3.5	−37.4±4.7**	−19.3±2.7**

Significant at *P≤0.05, **P≤0.01, and ***P≤0.001, (linear mixed effects model).

1 Absolute differences in stocks were calculated as means of rubber plantations within a cluster minus reference forest.

2 Relative differences in stocks were calculated as means of rubber plantations within a cluster minus reference forest divided by reference forest multiplied by 100.

**Figure 2:**
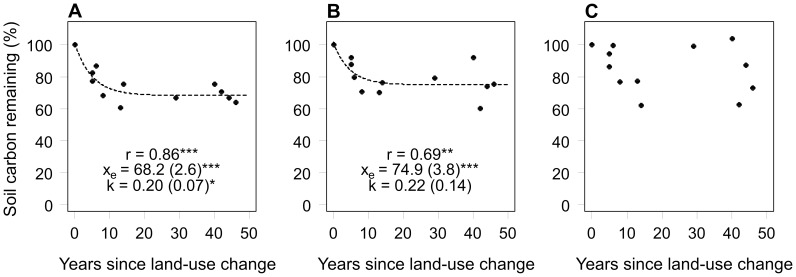
Soil carbon remaining after land-use change at (A) 0–0.15-m, (B) 0.15–0.3-m, and (C) 0.3–0.6-m depth. Soil carbon remaining is expressed as the proportion of soil carbon in rubber plantations relative to the secondary forest. The dashed lines represent fitted mono-exponential models (see Equation 2). r = Pearson’s correlation coefficient between observed and fitted values; k = decay rate (year^−1^) and X_e_ =  equilibrium ratio (%), and values in brackets are SE. Pearson’s r and parameter estimates are significant at *P≤0.05, **P≤0.01, and ***P≤0.001.

For the top 0.15-m depth, the proportion of carbon remaining in the soil exponentially decreased with the years since land-use change, as described by the mono-exponential model (Equation 2) (Fig. 2A). The largest decrease could be observed in the first 5 years following land-use change, when the soil carbon stocks had declined to approximately 80% of the original amount. A steady state was reached after approximately 20 years, when soil carbon stocks had declined to 68% of the original amount. At 0.15–0.3-m depth, soil carbon had the tendency to exponentially decrease with time but the estimated decay rate of the mono-exponential model was not significant; a steady state after approximately 20 years showed a soil carbon stock decline of 75% of the original amount (Fig. 2B). At 0.3–0.6-m depth (Fig. 2C), a mono-exponential trend was not detectable. Bi-exponential model (Equation 3) fitting resulted in insignificant decay rates for both the labile and stable soil carbon pool for all soil depths (data not shown). Furthermore, the fitted curves of the bi-exponential model and mono-exponential model were identical. Together these results indicate that the mono-exponential model was most suitable to describe the observed soil carbon changes in relation to the years since land-use change.

### Correlations of Soil Carbon Concentrations and Soil Carbon Stock Changes with Environmental Factors

In rubber plantations, soil carbon concentrations in the top 0.6-m of the soil showed positive correlations with altitude and with the sum of clay and silt content (and a negative correlation with sand content) (Table 4). However, at 0.15–0.3-m depth the correlation with the sum of silt and clay content was only marginally significant (P = 0.1). Rubber plantation age was not correlated to soil carbon concentrations in the top 0.6-m depth. However, for 0.9–1.2-m depth a positive correlation was observed between soil carbon concentrations and plantation age (spearman’s rho = 0.66, P≤0.05). In secondary forests, soil carbon concentrations at 0.15–0.3-m depth were positively correlated with the sum of clay and silt content and at 0.3–0.6-m depth with the total basal area of the forest (Table 4). The trends with soil texture and total basal area were also apparent at 0.6–0.9-m depth (data not shown). Relative differences in soil carbon stocks between rubber plantations and secondary forests in the top 0.15-m of the soil were negatively correlated with total basal area and rubber plantation age. In the top 0.6-m of the soil, relative differences in soil carbon stocks were positively correlated with altitude, but for the top 0.15-m of the soil this correlation was marginally significant (P = 0.06).

**Table 4:** Correlation coefficients^1^ of soil carbon concentrations and relative soil carbon stock differences^2^ with explanatory variables at three depths.

	Rubber plantation C (%) (n = 11)			Secondary forest C (%) (n = 7)			Relative soil C differences (%) (n = 9)		
Explanatory variable	0–0.15 m	0.15–0.3 m	0.3–0.6 m	0–0.15 m	0.15–0.3 m	0.3–0.6 m	0–0.15 m	0.15–0.3 m	0.3–0.6 m
Litter C stock (Mg ha^−1^)	0.21	0.39	0.40	−0.07	−0.18	0.36	−0.10	0.52	0.49
Litter C : N ratio	−0.40	−0.23	0.15	−0.43	−0.57	−0.11	−0.35	−0.25	0.04
Total basal area (m^2^ ha^−1^)	−0.39	−0.47	−0.24	0.18	0.57	0.89**	−0.64*	−0.55^†^	−0.48
Silt and clay (%)	0.72*	0.53^†^	0.66*	0.36	0.93**	0.61	0.13	0.20	0.49
Rubber plantation age (year)	−0.26	−0.34	−0.03	–	–	–	−0.65*	−0.46	−0.24
Slope (%)	−0.08	0.07	0.14	−0.46	−0.04	0.11	0.15	0.35	0.08
Altitude (m)	0.75**	0.76**	0.71*	−0.50	0.00	0.21	0.59^†^	0.70*	0.70*

1 Spearman rank correlation test, marginally significant at ^†^P≤0.1, and significant at *P≤0.05, and **P≤0.01.

2 Relative soil carbon stock differences were calculated as carbon stock in rubber plantation minus carbon stock of the reference secondary forest divided by carbon stock of the reference secondary forest multiplied by 100. Relative soil carbon stock differences were correlated with explanatory variables of the rubber plantations.

## Discussion

### Impact of Land-use Changes on Soil Carbon Stocks

Our findings of decreased soil carbon stocks under rubber plantations, not only in the top 0.6-m depth but also when considering the whole 1.2-m depth, are consistent with published studies of paired comparisons and chronosequences that all reported soil carbon losses following the conversion from primary or secondary forests to rubber plantations [14]–[16].

To estimate the effects of land-use changes on soil carbon stocks it is crucial to account for changes in soil bulk density. The importance of correction for bulk density changes has been emphasized by many authors [2], [6], [22], [30], but it was not applied in the published studies on forest-to-rubber plantation conversions [14]–[16]. We examined the effects of bulk density changes on the estimated land-use change effects for our own data and for the cited studies that reported data on depth, bulk density, and soil carbon concentrations [15], [16]. Comparison of corrected and uncorrected estimates showed that in these studies, not accounting for bulk density changes resulted in overestimations up to 5% and underestimations as high as 18% of the relative soil carbon stock difference (Table S2). Errors were greatest for the top 0.3-m depth. This comparison illustrates again that ignoring bulk density changes potentially causes large errors in land-use induced soil carbon stocks changes. For the following discussion of forest-to-rubber plantation conversions, we used the corrected values of relative soil carbon stock changes.

The only published study on secondary forest-to-rubber plantation conversion was conducted in Xishuangbanna on an Udic Ferrisol (Chinese classification system) [16]. Yang et al. [16] observed soil carbon losses (corrected for bulk density changes) of 24% in a 3-year-old rubber plantation and 21% in a 7-year-old rubber plantation in the top 0.6-m depth. The soil carbon stocks in rubber plantations and secondary forests reported by Yang et al. [16] were comparable to our estimated soil carbon stocks (Table 3). In Brazil, the conversion from primary forests to rubber plantations resulted in soil carbon losses of 21% down to 0.5-m depth in a 22-year-old rubber plantation on an Ultisol [14], and of 48% down to 1.0-m depth in a 17-year-old rubber plantation on an Oxisol [15]. The soil carbon losses observed by Araujo et al. [14] and Yang et al. [16] correspond well with our observed declines of 24% at 0–0.3-m depth and 21% at 0–0.6-m depth (Table 3). However, the 48% decline down to 1-m depth reported by Salimon et al. [15] is much larger than the 19% we observed down to 1.2-m depth.

Important methodological differences exist between our study and the cited studies. The large decrease in soil carbon stocks reported by Salimon et al. [15] should be interpreted with care, since in their study soil samples below 0.10-m depth were taken from the middle of each depth interval instead of sampling the entire depth interval, which can lead to inaccurate estimations of the soil carbon stocks. Furthermore, the studies from Araujo et al. [14] and Salimon et al. [15] consisted of only one forest and one rubber plantation and the study from Yang et al. [16] consisted of two rubber plantations and two forests. Results from such case studies with no or insufficient replications should not be extrapolated to a large scale. Finally, Yang et al. [16] and Salimon et al. [15] established in each land-use type one plot where they took no more than three replicate soil samples per depth interval.

At first sight, our observed decline in soil carbon stocks of 24±2.5% in the top 0.3-m depth seems to be much larger than the insignificant effects reported for the compiled studies of the whole tropics for forest-to-tropical tree plantation conversions [5]. However, analysis of the supplementary dataset from Powers et al. [5] revealed that 12 out of the 15 tree plantation types included in their meta-analysis showed soil carbon losses of 19±4.4% down to 0.3-m depth. The overall insignificant effect reported in their study resulted from the large number of observations from the other three plantation types (comprising 36% of all observations) that showed soil carbon accumulation, thereby offsetting the soil carbon losses. Our observed decline was thus within the same magnitude as carbon losses reported for many other tropical tree plantations. Taken as a whole, tropical tree plantations established after forest conversion appear to be more prone to soil carbon losses than previously reported.

The space-for-time substitution approach used in our study has as advantage that long-term soil carbon stock dynamics can be studied within a relatively short time period. However, this approach has also disadvantages: (i) It requires the untestable assumption that the land-use changes were random in the landscape regarding forest soil carbon stocks, and (ii) the spatial variation in other biophysical conditions of plots within a cluster (i.e. each cluster included a reference secondary forest and one to three rubber plantations) may interfere with land-use change effects and time trends. To deal with these limitations we carefully selected plots for comparison within a cluster and the clusters were replicated spatially. We included seven replicated clusters, which reduces the chance that soil carbon stock differences between rubber plantations and secondary forests are not due to land use.

### Soil Carbon Losses Related to Years since Land-use Change

The observed exponential decrease in soil carbon stocks in the top 0.15-m depth in rubber plantations with years since land-use change is similar to trends often reported for forest-to-agriculture conversions [31]–[33]. For our study, the potential drivers of the rapid soil carbon losses during the first five years following land-use change are: (i) soil disturbances during site preparation and terrace construction, which may accelerate soil surface erosion and increase soil carbon decomposition, and (ii) the sparse vegetation cover in young plantations, which reduces the soil carbon input and may change the microclimatic conditions, the latter could in turn result in enhanced soil carbon decomposition rates. The reduced soil carbon losses in older plantations may be explained by the denser vegetation cover, thereby increasing soil carbon input and soil stability. Management practices that are likely to affect the soil carbon balance during the entire rotation period of the rubber plantation are terrace maintenance, rubber tapping, fertilization and the removal of the vegetation understory.

Although a soil carbon steady state was reached approximately 20 years after conversion, we expect the land-use change induced soil carbon losses in this region to continue for a longer period of time. This is because the lifespan of rubber plantations in this region ranges between 30–50 years, and it is a common practice to establish new rubber plantations after felling the previous plantation. Site preparation for the new rubber trees involves the establishment of new terraces, which would be accompanied with a further decline in the soil carbon stock.

### Environmental Controls on Soil Carbon Concentrations and Soil Carbon Losses

In rubber plantations and secondary forests, the positive correlation of soil carbon concentrations with silt and clay content (Table 4) is consistent with literature and can be explained by the chemical and physical stabilization mechanisms of clay and silt particles [34]. The correlation between altitude and soil carbon concentrations, as we observed for rubber plantations, is often related to temperature effects. However, a temperature gradient is probably not the cause of the observed correlation, considering the relatively small altitude range of the sampled rubber plantations (700–830 m). Most likely the observed correlation reflects the marginally significant relationship between altitude and the sum of silt and clay content in the rubber plantations (data not shown). In secondary forests, the positive correlation of soil carbon concentrations with total basal area (which reflects forest productivity) suggests that increases of above-ground biomass could increase soil carbon input through increased input of leaf litter and root residues. This implies that in this region, restoration of the above-ground biomass in degraded secondary forests may increase soil carbon concentrations. The negative correlation between soil carbon stock differences and plantation age in 0–0.15-m depth attests that soil carbon stocks in rubber plantations progressively declined with increasing plantation age. This trend was also reflected in the negative correlation between soil carbon stock differences and total basal area due to the collinearity between plantation age and total basal area. The positive correlation between soil carbon stock differences and altitude may reflect the previously described soil texture gradient in rubber plantations with altitude.

### Changes in Above-ground Carbon Stocks versus Soil Carbon Losses

It is often assumed that soil carbon emissions arising from deforestation and forest degradation in the tropics are relatively small compared to above-ground carbon losses [2], [3], [35]. In our case, comparison of the observed soil carbon losses with the estimates of above-ground carbon changes based on regional data [36] reveals that forest conversion to rubber plantations had a much larger effect on soil carbon stocks than on above-ground carbon stocks. Above-ground carbon stock estimates for forests outside nature reserves range from 32.2–71.0 Mg C ha^−1^ with a mean of 53.2±2.1 Mg C ha^−1^ [36]. We assumed that these forests may reflect the conditions of our sampled forests, which were also situated outside nature reserves. For rubber plantations, means of above-ground carbon stock estimates range from 61.4 Mg C ha^−1^ for plantations <800-m altitude to 35.1 Mg C ha^−1^ for plantations between 800–1000-m altitude [36]. We used these estimates as our sampled plantations were situated between 700 and 830-m altitude. Together, this indicates that land-use change may results in above-ground carbon stock changes ranging from a loss of 18 Mg C ha^−1^ to an increase of 8 Mg C ha^−1^. These estimates are in agreement with our data on total basal area (Table 2), for which we did not detect differences between the total basal area of forests and rubber plantations. Such changes in above-ground carbon stocks were much lower than the soil carbon loss of 37.4±4.7 Mg C ha^−1^ for the entire 1.2-m depth.

### Consequences of Observed Soil Carbon Losses

We showed that the conversion from secondary forests to rubber plantations leads to diminishing soil carbon stocks and that this decline is much larger than the changes in above-ground carbon stocks. Given the clear pattern of our locally collected data, it is likely that in montane mainland southeast Asia on comparable soils, this land-use change may cause declines in the soil carbon stock, the magnitude of which will depend on site-specific biophysical conditions and management practices. The size of the observed losses has implications for the estimates of ecosystem carbon fluxes arising from land-use changes in the national inventories based on the IPCC guidelines. The IPCC tier 1 method does not include soil carbon fluxes for the forest-to-rubber plantation conversions [4], [17]. Our findings show that soil carbon changes need to be included to avoid possibly large errors in the estimates of the overall ecosystem carbon fluxes.

## Supporting Information

Table S1 **S1A. Site and soil characteristics of the secondary forest plots. S1B. Site and soil characteristics of the rubber plantation plots.**
(DOCX)

Table S2 **The effect^1^ of correction for bulk density changes on estimates of soil carbon stock changes.** Corrections for bulk density changes were applied for estimates of the soil carbon stocks in rubber plantations and for the estimates of absolute^2^ and relative^3^ differences in soil carbon stocks between rubber plantations and secondary forests for our own data^4^ (means ± SE), and for the cited studies that reported soil carbon concentration and bulk density data.
(DOCX)
