# Supplementary material for: Soil Carbon Stocks Decrease following Conversion of Secondary Forests to Rubber (Hevea brasiliensis) Plantations
Source: PLoS One. 2013 Jul 19;8(7):e69357. doi: 10.1371/journal.pone.0069357 (PMC3716606; doi:10.1371/journal.pone.0069357)
Supplement: Table S1 — S1A. Site and soil characteristics of the secondary forest plots. S1B. Site and soil characteristics of the rubber plantation plots. (DOCX) [file pone.0069357.s001.docx]

**Table S1A.** Site and soil characteristics of the secondary forest plots.

| **Cl.** | **Depth**  **(m)** | **Soil type^1^** | **Alt.**  **(m)** | **Asp.**  **(˚)** | **Sl.**  **(%)** | **Sand**  **(%)** | **S+C**  **(%)** | **BD**  **(g cm^-3^)** | **pH (H_2_O)** | **pH (KCl)** | **ECEC**  **(mmolc kg^-1^ soil)** | **BS**  **(%)** | **Soil C:N** | **Soil C**  **(%)** | **Soil C**  **(Mg ha^-1^)** |
| --- | --- | --- | --- | --- | --- | --- | --- | --- | --- | --- | --- | --- | --- | --- | --- |
| 1 | 0-0.15 | Fl CM (au, hu, hd, cr) | 828 | 187 | 61 | 52.1 | 47.9 | 1.3 | 5.3 | 4.1 | 61.1 | 58.4 | 14.6 | 2.6 | 51.2 |
| 1 | 0.15-0.3 | Fl CM (au, hu, hd, cr) | 828 | 187 | 61 | 52.2 | 47.8 | 1.4 | 5.4 | 4.0 | - | - | 11.9 | 2.0 | 43.1 |
| 1 | 0.3-0.6 | Fl CM (au, hu, hd, cr) | 828 | 187 | 61 | 48.6 | 51.4 | 1.5 | 5.4 | 4.0 | - | - | 8.7 | 1.2 | 55.9 |
| 1 | 0.6-0.9 | Fl CM (au, hu, hd, cr) | 828 | 187 | 61 | 40.7 | 59.3 | 1.5 | 4.9 | 3.9 | 57.9 | 19.1 | 6.5 | 0.7 | 30.8 |
| 1 | 0.9-1.2 | Fl CM (au, hu, hd, cr) | 828 | 187 | 61 | 38.8 | 61.2 | 1.5 | 4.9 | 3.9 | - | - | 6.0 | 0.5 | 22.1 |
| 2 | 0-0.15 | ha FR (hu, au, hd, ce) | 727 | 250 | 23 | 35.9 | 64.1 | 1.2 | 4.4 | 3.8 | 48.5 | 15.7 | 12.1 | 2.6 | 47.9 |
| 2 | 0.15-0.3 | ha FR (hu, au, hd, ce) | 727 | 250 | 23 | 46.5 | 53.5 | 1.3 | 4.7 | 3.9 | - | - | 11.2 | 2.0 | 38.5 |
| 2 | 0.3-0.6 | ha FR (hu, au, hd, ce) | 727 | 250 | 23 | 42.0 | 58.0 | 1.1 | 4.8 | 3.9 | - | - | 10.2 | 1.3 | 44.3 |
| 2 | 0.6-0.9 | ha FR (hu, au, hd, ce) | 727 | 250 | 23 | 41.8 | 58.2 | 1.4 | 4.5 | 3.9 | 35.6 | 12.5 | 9.0 | 0.8 | 33.1 |
| 2 | 0.9-1.2 | ha FR (hu, au, hd, ce) | 727 | 250 | 23 | 40.6 | 59.4 | 1.4 | 5.2 | 4.0 | - | - | 8.8 | 0.6 | 26.6 |
| 3 | 0-0.15 | ha FR (hu, au, hd, ce) | 742 | 340 | 30 | 34.0 | 66.0 | 0.9 | 4.3 | 3.8 | 59.3 | 15.1 | 11.8 | 3.0 | 41.2 |
| 3 | 0.15-0.3 | ha FR (hu, au, hd, ce) | 742 | 340 | 30 | 33.1 | 66.9 | 1.2 | 4.8 | 3.8 | - | - | 12.7 | 2.2 | 37.9 |
| 3 | 0.3-0.6 | ha FR (hu, au, hd, ce) | 742 | 340 | 30 | 33.1 | 66.9 | 1.1 | 4.7 | 3.9 | - | - | 11.9 | 1.5 | 48.9 |
| 3 | 0.6-0.9 | ha FR (hu, au, hd, ce) | 742 | 340 | 30 | 31.7 | 68.3 | 1.2 | 4.9 | 3.9 | 38.6 | 7.2 | 9.9 | 0.9 | 31.7 |
| 3 | 0.9-1.2 | ha FR (hu, au, hd, ce) | 742 | 340 | 30 | 27.6 | 72.4 | 1.2 | 5.0 | 3.9 | - | - | 9.6 | 0.8 | 28.8 |
| 4 | 0-0.15 | vt FR (hu, au, hd, ce) | 797 | 130 | 40 | 37.3 | 62.7 | 0.9 | 4.8 | 3.9 | 44.4 | 28.4 | 11.0 | 2.4 | 33.4 |
| 4 | 0.15-0.3 | vt FR (hu, au, hd, ce) | 797 | 130 | 40 | 38.1 | 61.9 | 1.1 | 5.0 | 4.0 | - | - | 11.1 | 2.0 | 34.2 |
| 4 | 0.3-0.6 | vt FR (hu, au, hd, ce) | 797 | 130 | 40 | 35.3 | 64.7 | 1.1 | 4.8 | 4.0 | - | - | 11.3 | 1.6 | 55.4 |
| 4 | 0.6-0.9 | vt FR (hu, au, hd, ce) | 797 | 130 | 40 | 35.1 | 64.9 | 1.3 | 5.3 | 4.0 | 28.2 | 12.1 | 9.6 | 0.8 | 29.5 |
| 4 | 0.9-1.2 | vt FR (hu, au, hd, ce) | 797 | 130 | 40 | 31.9 | 68.1 | 1.4 | 5.2 | 4.1 | - | - | 9.7 | 0.7 | 29.6 |
| 5 | 0-0.15 | ac FR (hu, au, hd, ce) | 750 | 250 | 17 | 45.2 | 54.8 | 1.0 | 4.6 | 3.8 | 59.2 | 15.0 | 12.8 | 3.3 | 49.5 |
| 5 | 0.15-0.3 | ac FR (hu, au, hd, ce) | 750 | 250 | 17 | 36.1 | 63.9 | 1.2 | 4.8 | 3.9 | - | - | 12.7 | 2.1 | 36.9 |
| 5 | 0.3-0.6 | ac FR (hu, au, hd, ce) | 750 | 250 | 17 | 32.8 | 67.2 | 1.2 | 4.5 | 4.0 | - | - | 12.1 | 1.4 | 53.6 |
| 5 | 0.6-0.9 | ac FR (hu, au, hd, ce) | 750 | 250 | 17 | 34.3 | 65.7 | 1.4 | 4.6 | 4.0 | 37.5 | 7.2 | 8.9 | 0.7 | 31.2 |
| 5 | 0.9-1.2 | ac FR (hu, au, hd, ce) | 750 | 250 | 17 | 34.5 | 65.5 | 1.4 | 5.3 | 3.9 | - | - | 8.5 | 0.6 | 26.0 |
| 6 | 0-0.15 | ha FR (hu, au, hd, ce) | 790 | 85 | 30 | 19.3 | 80.7 | 1.1 | 4.8 | 3.9 | 56.5 | 15.5 | 13.5 | 2.9 | 47.7 |
| 6 | 0.15-0.3 | ha FR (hu, au, hd, ce) | 790 | 85 | 30 | 16.6 | 83.4 | 1.2 | 4.6 | 3.9 | - | - | 14.5 | 2.6 | 45.5 |
| 6 | 0.3-0.6 | ha FR (hu, au, hd, ce) | 790 | 85 | 30 | 15.3 | 84.7 | 1.2 | 4.9 | 3.9 | - | - | 12.1 | 1.5 | 52.2 |
| 6 | 0.6-0.9 | ha FR (hu, au, hd, ce) | 790 | 85 | 30 | 14.8 | 85.2 | 1.3 | 5.2 | 4.0 | 35.2 | 9.8 | 9.4 | 0.8 | 32.6 |
| 6 | 0.9-1.2 | ha FR (hu, au, hd, ce) | 790 | 85 | 30 | 12.4 | 87.6 | 1.2 | 5.7 | 4.1 | - | - | 7.8 | 0.7 | 24.6 |
| 7 | 0-0.15 | vt FR (hu, au, hd, ce) | 768 | 60 | 42 | 18.9 | 81.1 | 0.8 | 4.7 | 3.9 | 59.5 | 20.4 | 14.0 | 3.2 | 36.4 |
| 7 | 0.15-0.3 | vt FR (hu, au, hd, ce) | 768 | 60 | 42 | 18.3 | 81.7 | 1.1 | 4.6 | 3.9 | - | - | 13.1 | 2.1 | 36.3 |
| 7 | 0.3-0.6 | vt FR (hu, au, hd, ce) | 768 | 60 | 42 | 18.0 | 82.0 | 1.2 | 4.7 | 3.9 | - | - | 12.6 | 1.5 | 54.1 |
| 7 | 0.6-0.9 | vt FR (hu, au, hd, ce) | 768 | 60 | 42 | 12.4 | 87.6 | 1.2 | 4.7 | 4.0 | 25.5 | 11.4 | 12.7 | 1.6 | 57.1 |
| 7 | 0.9-1.2 | vt FR (hu, au, hd, ce) | 768 | 60 | 42 | 13.4 | 86.6 | 1.2 | 4.7 | 4.2 | - | - | 8.6 | 0.7 | 24.4 |

*Cl. = Cluster, Alt. = Altitude, Sl. = Slope, S+C= Silt and Clay concentration, BD = Bulk Density, ECEC = Effective Cation Exchange Capacity, BS = Base Saturation.*

*^1^Codes for Reference Soil Group [18]: CM = Cambisol, FR = Ferralsol, and codes for qualifiers: ac = Acric, au = Alumic, ce = Clayic, cr = Chromic, dy = Dystic, dyo = Orthodystic, ec = Escalic, fl = Ferralic, flh = Hyperferralic, ha = Haplic, hd = Hyperdystic, hu = Humic, vt = Vetic, xa = Xanthic.*

**Table S1B.** Site and soil characteristics of the rubber plantation plots.

| **Cl.** | **Age**  **(y)** | **Depth**  **(m)** | **Soil type^1^** | **Alt.**  **(m)** | **Asp.**  **(˚)** | **Sl.**  **(%)** | **Sand**  **(%)** | **S+C**  **(%)** | **BD**  **(gcm^-3^)** | **pH (H_2_O)** | **pH (KCl)** | **ECEC**  **(mmol c kg^-1^ soil)** | **BS**  **(%)** | **Soil C:N** | **Soil C**  **(%)** | **Soil C^2^**  **(Mg ha^-1^)** |
| --- | --- | --- | --- | --- | --- | --- | --- | --- | --- | --- | --- | --- | --- | --- | --- | --- |
| 1 | 5 | 0-0.15 | flh CM (au, hd, ec) | 797 | 228 | 65 | 47.7 | 52.3 | 1.3 | 5.5 | 4.1 | 41.7 | 46.7 | 12.0 | 2.0 | 39.6 |
| 1 | 5 | 0.15-0.3 | flh CM (au, hd, ec) | 797 | 228 | 65 | 48.1 | 51.9 | 1.0 | 5.4 | 4.0 | - | - | 12.1 | 1.9 | 39.8 |
| 1 | 5 | 0.3-0.6 | flh CM (au, hd, ec) | 797 | 228 | 65 | 47.2 | 52.9 | 1.4 | 5.4 | 4.0 | - | - | 10.0 | 1.2 | 52.8 |
| 1 | 5 | 0.6-0.9 | flh CM (au, hd, ec) | 797 | 228 | 65 | 44.3 | 55.7 | 1.6 | 5.3 | 4.0 | 31.9 | 12.7 | 7.1 | 0.4 | 20.6 |
| 1 | 5 | 0.9-1.2 | flh CM (au, hd, ec) | 797 | 228 | 65 | 46.6 | 53.4 | 1.4 | 5.3 | 3.9 | - | - | 5.6 | 0.3 | 13.6 |
| 2 | 8 | 0-0.15 | vt ac FR (au, hd, ce, ec) | 711 | 330 | 22 | 43.4 | 56.6 | 1.0 | 4.7 | 3.7 | 38.9 | 12.0 | 10.6 | 1.8 | 32.8 |
| 2 | 8 | 0.15-0.3 | vt ac FR (au, hd, ce, ec) | 711 | 330 | 22 | 41.9 | 58.1 | 1.0 | 4.6 | 3.8 | - | - | 10.2 | 1.4 | 27.3 |
| 2 | 8 | 0.3-0.6 | vt ac FR (au, hd, ce, ec) | 711 | 330 | 22 | 38.1 | 61.9 | 1.1 | 4.6 | 3.9 | - | - | 10.1 | 1.0 | 34.0 |
| 2 | 8 | 0.6-0.9 | vt ac FR (au, hd, ce, ec) | 711 | 330 | 22 | 34.9 | 65.1 | 1.2 | 5.3 | 4.0 | 26.1 | 10.7 | 9.2 | 0.7 | 28.8 |
| 2 | 8 | 0.9-1.2 | vt ac FR (au, hd, ce, ec) | 711 | 330 | 22 | 36.9 | 63.1 | 1.3 | 5.0 | 4.0 | - | - | 8.2 | 0.5 | 22.4 |
| 3 | 5 | 0-0.15 | ha FR (hu, au, hd, ce, ec) | 767 | 330 | 39 | 39.9 | 60.1 | 0.9 | 4.7 | 3.8 | 52.6 | 13.9 | 13.3 | 2.5 | 34.1 |
| 3 | 5 | 0.15-0.3 | ha FR (hu, au, hd, ce, ec) | 767 | 330 | 39 | 38.1 | 61.9 | 1.1 | 4.6 | 3.8 | - | - | 13.5 | 1.9 | 33.3 |
| 3 | 5 | 0.3-0.6 | ha FR (hu, au, hd, ce, ec) | 767 | 330 | 39 | 37.4 | 62.6 | 1.2 | 4.9 | 3.8 | - | - | 12.3 | 1.3 | 42.4 |
| 3 | 5 | 0.6-0.9 | ha FR (hu, au, hd, ce, ec) | 767 | 330 | 39 | 38.3 | 61.7 | 1.4 | 4.7 | 3.9 | 30.7 | 5.9 | 9.4 | 0.7 | 26.1 |
| 3 | 5 | 0.9-1.2 | ha FR (hu, au, hd, ce, ec) | 767 | 330 | 39 | 35.9 | 64.1 | 1.4 | 4.8 | 4.0 | - | - | 8.8 | 0.7 | 24.0 |
| 3 | 44 | 0-0.15 | vt ac FR (au, hd, ce, ec) | 730 | 310 | 39 | 26.0 | 74.0 | 1.2 | 4.8 | 3.9 | 44.6 | 29.7 | 11.1 | 2.0 | 27.6 |
| 3 | 44 | 0.15-0.3 | vt ac FR (au, hd, ce, ec) | 730 | 310 | 39 | 23.2 | 76.8 | 1.2 | 4.9 | 3.9 | - | - | 11.3 | 1.6 | 28.1 |
| 3 | 44 | 0.3-0.6 | vt ac FR (au, hd, ce, ec) | 730 | 310 | 39 | 20.2 | 79.8 | 1.2 | 4.9 | 4.0 | - | - | 10.6 | 1.3 | 42.6 |
| 3 | 44 | 0.6-0.9 | vt ac FR (au, hd, ce, ec) | 730 | 310 | 39 | 22.7 | 77.3 | 1.2 | 5.0 | 4.1 | 26.6 | 14.0 | 9.4 | 0.9 | 33.2 |
| 3 | 44 | 0.9-1.2 | vt ac FR (au, hd, ce, ec) | 730 | 310 | 39 | 20.5 | 79.5 | 1.1 | 5.2 | 4.3 | - | - | 8.1 | 0.7 | 24.8 |
| 3 | 13 | 0-0.15 | ha FR (au, hd, ce, xa, ec) | 697 | 312 | 37 | 44.6 | 55.4 | 1.2 | 4.7 | 3.8 | 41.1 | 16.4 | 12.3 | 1.8 | 25.0 |
| 3 | 13 | 0.15-0.3 | ha FR (au, hd, ce, xa, ec) | 697 | 312 | 37 | 41.7 | 58.3 | 1.3 | 4.7 | 3.3 | - | - | 12.2 | 1.5 | 26.6 |
| 3 | 13 | 0.3-0.6 | ha FR (au, hd, ce, xa, ec) | 697 | 312 | 37 | 39.6 | 60.4 | 1.3 | 4.6 | 3.9 | - | - | 11.0 | 1.1 | 37.9 |
| 3 | 13 | 0.6-0.9 | ha FR (au, hd, ce, xa, ec) | 697 | 312 | 37 | 39.3 | 60.7 | 1.3 | 4.9 | 4.1 | 28.8 | 7.5 | 8.8 | 0.6 | 20.8 |
| 3 | 13 | 0.9-1.2 | ha FR (au, hd, ce, xa, ec) | 697 | 312 | 37 | 37.2 | 62.8 | 1.3 | 4.9 | 4.0 | - | - | 8.6 | 0.5 | 18.7 |
| 4 | 42 | 0-0.15 | ac FR (au, dy, ce, ec) | 743 | 123 | 43 | 43.1 | 56.9 | 1.3 | 4.9 | 3.9 | 40.9 | 30.4 | 11.6 | 1.7 | 23.6 |
| 4 | 42 | 0.15-0.3 | ac FR (au, dy, ce, ec) | 743 | 123 | 43 | 36.1 | 63.9 | 1.1 | 4.8 | 3.9 | - | - | 10.9 | 1.2 | 20.6 |
| 4 | 42 | 0.3-0.6 | ac FR (au, dy, ce, ec) | 743 | 123 | 43 | 28.9 | 71.1 | 1.3 | 5.0 | 4.0 | - | - | 10.3 | 1.0 | 34.6 |
| 4 | 42 | 0.6-0.9 | ac FR (au, dy, ce, ec) | 743 | 123 | 43 | 21.4 | 78.7 | 1.2 | 5.5 | 4.1 | - | - | 7.8 | 0.6 | 21.0 |
| 4 | 42 | 0.9-1.2 | ac FR (au, dy, ce, ec) | 743 | 123 | 43 | 19.1 | 80.9 | 1.2 | 5.4 | 4.2 | - | - | 7.7 | 0.6 | 24.0 |
| 4 | 14 | 0-0.15 | ha FR (au, hd, ce, ec) | 717 | 104 | 43 | 40.2 | 59.8 | 1.1 | 4.8 | 3.8 | 43.8 | 10.7 | 11.2 | 1.8 | 25.3 |
| 4 | 14 | 0.15-0.3 | ha FR (au, hd, ce, ec) | 717 | 104 | 43 | 38.2 | 61.8 | 1.1 | 4.9 | 3.8 | - | - | 10.2 | 1.6 | 26.1 |
| 4 | 14 | 0.3-0.6 | ha FR (au, hd, ce, ec) | 717 | 104 | 43 | 37.2 | 62.8 | 1.2 | 4.9 | 3.8 | - | - | 9.6 | 1.0 | 34.4 |
| 4 | 14 | 0.6-0.9 | ha FR (au, hd, ce, ec) | 717 | 104 | 43 | 34.9 | 65.1 | 1.4 | 5.1 | 3.9 | 33.5 | 7.5 | 8.2 | 0.6 | 21.6 |
| 4 | 14 | 0.9-1.2 | ha FR (au, hd, ce, ec) | 717 | 104 | 43 | 36.3 | 63.7 | 1.4 | 5.2 | 3.9 | - | - | 7.6 | 0.5 | 22.1 |
| 5 | 46 | 0-0.15 | vt ac FR (au, hd, ce, ec) | 742 | 253 | 25 | 23.2 | 76.8 | 1.3 | 4.7 | 3.9 | 45.5 | 29.1 | 12.1 | 2.1 | 31.8 |
| 5 | 46 | 0.15-0.3 | vt ac FR (au, hd, ce, ec) | 742 | 253 | 25 | 26.8 | 73.2 | 1.2 | 4.9 | 3.9 | - | - | 11.2 | 1.6 | 27.9 |
| 5 | 46 | 0.3-0.6 | vt ac FR (au, hd, ce, ec) | 742 | 253 | 25 | 20.5 | 79.5 | 1.3 | 5.1 | 4.0 | - | - | 10.2 | 1.1 | 39.2 |
| 5 | 46 | 0.6-0.9 | vt ac FR (au, hd, ce, ec) | 742 | 253 | 25 | 17.6 | 82.4 | 1.3 | 5.0 | 4.1 | 26.9 | 12.2 | 9.3 | 0.9 | 37.5 |
| 5 | 46 | 0.9-1.2 | vt ac FR (au, hd, ce, ec) | 742 | 253 | 25 | 15.4 | 84.6 | 1.3 | 5.2 | 4.1 | - | - | 8.2 | 0.7 | 30.3 |
| 6 | 6 | 0-0.15 | vt FR (hu,au, hd, xa, ec) | 836 | 80 | 23 | 16.0 | 84.0 | 0.8 | 4.7 | 3.8 | 53.5 | 13.5 | 13.1 | 2.5 | 41.5 |
| 6 | 6 | 0.15-0.3 | vt FR (hu,au, hd, xa, ec) | 836 | 80 | 23 | 14.1 | 85.9 | 1.2 | 4.7 | 3.8 | - | - | 12.9 | 2.1 | 36.4 |
| 6 | 6 | 0.3-0.6 | vt FR (hu,au, hd, xa, ec) | 836 | 80 | 23 | 12.0 | 88.0 | 1.1 | 4.7 | 3.8 | - | - | 12.0 | 1.5 | 52.1 |
| 6 | 6 | 0.6-0.9 | vt FR (hu,au, hd, xa, ec) | 836 | 80 | 23 | 12.9 | 87.1 | 1.2 | 4.8 | 3.9 | 32.0 | 5.4 | 8.7 | 0.8 | 32.5 |
| 6 | 6 | 0.9-1.2 | vt FR (hu,au, hd, xa, ec) | 836 | 80 | 23 | 16.4 | 83.7 | 1.3 | 4.9 | 4.0 | - | - | 7.7 | 0.7 | 24.7 |
| 7 | 40 | 0-0.15 | vt ac FR (hu, au, dyo, ce, ec) | 784 | 60 | 40 | 14.5 | 85.5 | 0.9 | 4.6 | 3.9 | 51.4 | 16.4 | 12.6 | 2.4 | 27.4 |
| 7 | 40 | 0.15-0.3 | vt ac FR (hu, au, dyo, ce, ec) | 784 | 60 | 40 | 11.6 | 88.4 | 1.1 | 4.8 | 3.9 | - | - | 14.3 | 2.0 | 33.4 |
| 7 | 40 | 0.3-0.6 | vt ac FR (hu, au, dyo, ce, ec) | 784 | 60 | 40 | 10.9 | 89.1 | 1.2 | 4.9 | 4.0 | - | - | 13.6 | 1.6 | 56.3 |
| 7 | 40 | 0.6-0.9 | vt ac FR (hu, au, dyo, ce, ec) | 784 | 60 | 40 | 10.9 | 89.2 | 1.1 | 5.1 | 4.2 | 22.3 | 22.0 | 11.5 | 0.8 | 29.5 |
| 7 | 40 | 0.9-1.2 | vt ac FR (hu, au, dyo, ce, ec) | 784 | 60 | 40 | 42.6 | 57.5 | 1.1 | 5.1 | 4.2 | - | - | 9.6 | 0.7 | 24.9 |
| 7 | 29 | 0-0.15 | Ac FR (hu, au, dy, ce, ec) | 813 | 70 | 49 | 19.3 | 80.7 | 1.1 | 4.9 | 4.0 | 56.4 | 49.3 | 13.9 | 2.1 | 24.4 |
| 7 | 29 | 0.15-0.3 | Ac FR (hu, au, dy, ce, ec) | 813 | 70 | 49 | 15.3 | 84.7 | 1.1 | 5.0 | 3.9 | - | - | 13.9 | 1.7 | 28.8 |
| 7 | 29 | 0.3-0.6 | Ac FR (hu, au, dy, ce, ec) | 813 | 70 | 49 | 14.2 | 85.8 | 1.2 | 5.0 | 3.9 | - | - | 13.8 | 1.5 | 53.6 |
| 7 | 29 | 0.6-0.9 | Ac FR (hu, au, dy, ce, ec) | 813 | 70 | 49 | 12.0 | 88.0 | 1.2 | 5.3 | 4.1 | - | - | 11.5 | 1.0 | 36.6 |
| 7 | 29 | 0.9-1.2 | Ac FR (hu, au, dy, ce, ec) | 813 | 70 | 49 | 14.0 | 86.0 | 1.3 | 5.3 | 4.1 | - | - | 10.3 | 0.7 | 25.1 |

*Cl. = Cluster, Alt. = Altitude, Sl. = Slope, S+C= Silt and Clay concentration, BD = Bulk Density, ECEC = Effective Cation Exchange Capacity, BS = Base Saturation.*

*^1^Codes for Reference Soil Group [18]: CM = Cambisol, FR = Ferralsol, and Codes for Qualifiers: ac = Acric, au = Alumic, ce = Clayic, cr = Chromic, dy = Dystic, dyo = Orthodystic, ec = Escalic, fl = Ferralic, flh = Hyperferralic, ha = Haplic, hd = Hyperdystic, hu = Humic, vt = Vetic, xa = Xanthic.*

*^2^Soil C stocks in rubber plantations were calculated with the bulk density data from the secondary forest (see methods).*
