# Supplementary material for: Soil Carbon Stocks Decrease following Conversion of Secondary Forests to Rubber (Hevea brasiliensis) Plantations
Source: PLoS One. 2013 Jul 19;8(7):e69357. doi: 10.1371/journal.pone.0069357 (PMC3716606; doi:10.1371/journal.pone.0069357)
Supplement: Table S2 — The effect1 of correction for bulk density changes on estimates of soil carbon stock changes. Corrections for bulk density changes were applied for estimates of the soil carbon stocks in rubber plantations and for the estimates of absolute2 and relative3 differences in soil carbon stocks between rubber plantations and secondary forests for our own data4 (means ± SE), and for the cited studies that reported soil carbon concentration and bulk density data. (DOCX) [file pone.0069357.s002.docx]

***Table S2.*** *The effect^1^ of correction for bulk density changes on estimates of soil carbon stock changes. Corrections for bulk density changes were applied for estimates of the soil carbon stocks in rubber plantations and for the estimates of absolute^2^ and relative^3^ differences in soil carbon stocks between rubber plantations and secondary forests for our own data^4^ (means ± SE), and for the cited studies that reported soil carbon concentration and bulk density data.*

|  |  |  | **Soil C stock (Mg ha^-1^)** | | | **Abs. soil C stock difference**  **(Mg ha^-1^)** | | **Rel. soil C stock difference**  **(%)** | |  |  |
| --- | --- | --- | --- | --- | --- | --- | --- | --- | --- | --- | --- |
| **Country** | **Age (year)** | **Depth (m)** | **Forest** | **Rubber Pl.**  **(Without BD correction)** | **Rubber Pl.**  **(With BD correction)** | **Without BD correction** | **With BD correction** | **Without BD correction** | **With BD correction** | **Effect (%)** | **Source** |
| China | 5-46 | 0-0.15 | 43.9 ± 2.6 | 33.7 ± 1.2 | 30.3 ± 1.9 | -10.0 ± 2.7 | -11.8 ± 1.4 | -21.2 ± 5.5 | -26.9 ± 2.8 | -6 | Own data |
| China | 5-46 | 0.15-0.3 | 38.9 ± 1.5 | 28.5 ± 1.4 | 29.8 ± 1.6 | -10.4 ± 1.5 | -8.2 ± 1.1 | -26.7 ± 3.9 | -21.4 ± 3.2 | 5 | Own data |
| China | 5-46 | 0.3-0.6 | 52.0 ± 1.6 | 45.4 ± 2.1 | 43.6 ± 2.6 | -7.0 ± 2.5 | -8.0 ± 3.0 | -13.6 ± 4.8 | -15.4 ± 5.6 | -2 | Own data |
| China | 5-46 | 0.6-0.9 | 35.2 ± 3.7 | 27.9 ± 1.8 | 28.0 ± 1.9 | -7.3 ± 3.5 | -6.5 ± 3.6 | -18.2 ± 7.3 | -16.0 ± 8.0 | 2 | Own data |
| China | 5-46 | 0.9-1.2 | 26.0 ± 1.0 | 22.8 ± 1.2 | 23.1 ± 1.3 | -3.6 ± 1.7 | -2.9 ± 1.8 | -13.7 ± 6.7 | -11.2 ± 7.0 | 2 | Own data |
| China | 5-46 | 0-1.2 | 196.0 ± 3.5 | 158.3 ± 5.6 | 154.9 ± 6.2 | -38.3 ± 5.5 | -37.4 ± 4.7 | -19.7 ± 2.9 | -19.3 ± 2.7 | 0 | Own data |
| China | 3 | 0-0.2 | 68.7 | 45.3 | 41.9 | -23.4 | -26.8 | -34 | -39 | -5 | Yang et al. [16] |
| China | 3 | 0.2-0.4 | 46.3 | 39.4 | 37.7 | -6.8 | -8.6 | -15 | -19 | -4 | Yang et al. [16] |
| China | 3 | 0.4-0.6 | 35.9 | 35.3 | 34.9 | -0.6 | -1.0 | -2 | -3 | -1 | Yang et al. [16] |
| China | 3 | 0-0.6 | 150.9 | 120.1 | 114.6 | -30.8 | -36.3 | -20 | -24 | -4 | Yang et al. [16] |
| China | 7 | 0-0.2 | 68.7 | 52.5 | 47.9 | -16.2 | -20.9 | -24 | -30 | -7 | Yang et al. [16] |
| China | 7 | 0.2-0.4 | 46.3 | 40.3 | 38.2 | -6.0 | -8.0 | -13 | -17 | -4 | Yang et al. [16] |
| China | 7 | 0.4-0.6 | 35.9 | 34.2 | 33.6 | -1.7 | -2.3 | -5 | -7 | -2 | Yang et al. [16] |
| China | 7 | 0-0.6 | 150.9 | 127.0 | 119.6 | -23.9 | -31.2 | -16 | -21 | -5 | Yang et al. [16] |
| Brazil | 17 | 0-0.05 | 9.1 | 6.8 | 5.1 | -2.3 | -4.0 | -25 | -43 | -18 | Salimon et al. [15] |
| Brazil | 17 | 0.05-0.1 | 7.5 | 4.7 | 3.7 | -2.8 | -3.8 | -37 | -50 | -13 | Salimon et al. [15] |
| Brazil | 17 | 0.1-0.175 | 9.3 | 5.5 | 4.4 | -3.8 | -4.9 | -41 | -52 | -12 | Salimon et al. [15] |
| Brazil | 17 | 0.175-0.275 | 10.6 | 7.5 | 7.2 | -3.1 | -3.4 | -29 | -32 | -3 | Salimon et al. [15] |
| Brazil | 17 | 0.275-0.375 | 9.3 | 4.9 | 4.3 | -4.4 | -5.0 | -47 | -54 | -7 | Salimon et al. [15] |
| Brazil | 17 | 0.375-0.475 | 9.8 | 4.8 | 4.6 | -5.0 | -5.2 | -51 | -53 | -2 | Salimon et al. [15] |
| Brazil | 17 | 0.475-0.625 | 11.3 | 7.9 | 7.3 | -3.4 | -4.0 | -30 | -36 | -6 | Salimon et al. [15] |
| Brazil | 17 | 0.625-0.825 | 14.4 | 6.8 | 6.3 | -7.6 | -8.1 | -53 | -57 | -4 | Salimon et al. [15] |
| Brazil | 17 | 0.825-1.0 | 14.5 | 7.3 | 6.8 | -7.2 | -7.7 | -50 | -53 | -3 | Salimon et al. [15] |
| Brazil | 17 | 0-1.0 | 95.8 | 56.2 | 49.7 | -39.6 | -46.1 | -41 | -48 | -7 | Salimon et al. [15] |

*Rubber Pl. = Rubber plantation, Abs. = Absolute, Rel. = Relative, BD = Bulk Density*

*^1^Effect was calculated as the relative stock differences corrected for BD changes minus the relative stock differences not corrected for BD changes.*

*^2^Absolute differences in stocks were calculated as rubber plantations minus reference forest.*

*^3^Relative differences in stocks were calculated as rubber plantations minus reference forest divided by reference forest multiplied by 100.*

*^4^For our own data, the absolute and relative differences in soil C stocks were based on comparison of the means of rubber plantations with the reference forest.*
